# The Paradox of Fiction Revisited—Improvised Fictional and Real-Life Social Rejections Evoke Associated and Relatively Similar Psychophysiological Responses

**DOI:** 10.3390/brainsci11111463

**Published:** 2021-11-05

**Authors:** Sirke Seppänen, Tapio Toivanen, Tommi Makkonen, Iiro P. Jääskeläinen, Kaisa Tiippana

**Affiliations:** 1 Faculty of Educational Sciences, University of Helsinki, P.O. Box 8, FI-00014 University of Helsinki, Finland; tapio.toivanen@helsinki.fi; 2 Cicero Learning, Faculty of Educational Sciences, University of Helsinki, P.O. Box 9, FI-00014 University of Helsinki, Finland; 3 Cognitive Brain Research Unit, Department of Psychology and Logopedics, Faculty of Medicine, University of Helsinki, P.O. Box 21, FI-00014 University of Helsinki, Finland; tommi.makkonen@helsinki.fi; 4 Brain and Mind Laboratory, Department of Neuroscience and Biomedical Engineering, Aalto University, FI-00076 Aalto, Finland; iiro.jaaskelainen@aalto.fi; 5 International Laboratory of Social Neurobiology, Institute of Cognitive Neuroscience, National Research University Higher School of Economics, 101000 Moscow, Russia; 6 Department of Psychology and Logopedics, Faculty of Medicine, University of Helsinki, P.O. Box 21, FI-00014 University of Helsinki, Finland; kaisa.tiippana@helsinki.fi

**Keywords:** improvisation, social rejection, theatre-based practices, experiential learning, paradox of fiction, social interaction

## Abstract

Theatre-based practices, such as improvisation, are frequently applied to simulate everyday social interactions. Although the improvisational context is acknowledged as fictional, realistic emotions may emerge, a phenomenon labelled the ‘paradox of fiction’. This study investigated how manipulating the context (real-life versus fictional) modulates psychophysiological reactivity to social rejection during dyadic interactions. We measured psychophysiological responses elicited during real-life (interview) and fictional (improvisation exercises) social rejections. We analysed the heart rate (HR), skin conductance, facial muscle activity, and electrocortical activity (electroencephalographic (EEG) alpha asymmetry) of student teachers (*N* = 39) during various social rejections (devaluing, interrupting, nonverbal rejection). All social rejections evoked negative EEG alpha asymmetry, a measure reflecting behavioural withdrawal motivation. Psychophysiological responses during real-life and fictional rejections correlated, and rejection type modified the responses. When comparing responses across all rejection types, facial muscle activity and EEG alpha asymmetry did not differ between real-life and fictional rejections, whereas HR decelerated and skin conductance increased during fictional rejections. These findings demonstrate that regardless of cognitive awareness of fictionality, relatively subtle social rejections elicited psychophysiological reactivity indicating emotional arousal and negative valence. These findings provide novel, biological evidence for the application of theatre-based improvisation to studying experientially everyday social encounters.

## 1. Introduction

‘It was interesting to perceive how real the feelings of becoming rejected were during the negation exercises. No matter how aware I was that the conversation partners were supposed to behave rudely and that they would not behave like that in a real-life interaction, I found myself feeling slightly annoyed and taken aback that they were not interested in what I was saying’. This reflection from one student teacher describes their experiences from emotional moments during a theatre-based improvisation course, carried out as a part of a teacher education programme at the University of Helsinki in 2017 and 2018. The course aimed to support student teachers’ professional interaction and performance skills. At times, engagement in improvisation exercises was so intense that participants had to ‘shake off’ their negative sensations following social rejection exercises. Fascinatingly, entirely fictional improvisation exercises prompted genuine emotional responses. The student teacher cited was certainly aware of the fictionality of both the contents and context of the exercise, since they participated in a role-play in a drama classroom knowing the purpose of the simulation. However, reality is not a necessary condition for eliciting genuine emotional responses, as suggested by the ‘paradox of fiction’ [1,2,3]. The paradox of fiction refers to emotions we feel towards fictional characters and events even when we know they do not exist. Other participants of this course described similar sensations, as did numerous participants from previous improvisation courses conducted by the same instructor (first author) over a decade. These apparently strong, frequently occurring, and clearly physical reactions inspired this study, which explores the psychophysiology of social rejections during improvisation exercises. Through this study, we attempt to answer the following question: if the student teachers genuinely responded emotionally to a fictional simulation of a social interaction, was the physiological arousal of the emotion genuine as well?

Several studies report stronger emotional and psychophysiological responses to real-life relative to fictional stimuli. In an early study by Geen [4], participants watched videotaped fight scenes labelled real or fictional, while their blood pressure and skin conductance were measured to indicate an emotional arousal. Observing real-labelled violence aroused participants more relative to the fictional counterpart. More recently, Mocaiber et al. [5] studied the effect of real and fictional affective images on reaction time and electrocortical activity (late positive potential or LPP) in the context of implicit emotion regulation. They found that both reaction time and LPP amplitude were attenuated during fictional compared to real-life contexts, suggesting that affective processing is flexible and highly context-dependent. Moreover, Mendelson and Papacharissi [6] observed stronger emotional responses to photographs labelled real relative to fictional. These findings suggest that cognitive strategies (that is, ‘I know this is not real’) are capable of downplaying emotions by diminishing the relevance of emotional stimuli. In research on brain networks, De Borst et al. [7] showed in their study of mental imagery that a machine learning algorithm could determine from cinematographers’ brain activation whether they imagined a documentary (i.e., real) or a fictional film clip.

However, the literature also provides evidence on the equivalent behavioural, emotional, and neuronal responses in real-life and fictional contexts. For example, social ostracism need not result from real people. During a ball-tossing computer game, rejection resulted in hurt feelings [8,9], even though players knew they were competing against a computer [10]. Identical film clips labelled either as fiction or based on real events elicited equivalent levels of self-reported sadness and anxiety [11]. Additionally, in virtual reality contexts, exposure to real and virtual food elicited similar emotional responses and physiological arousal measured through heart rate (HR) and skin conductance [12]. Another virtual reality study identified life-like behavioural and psychophysiological responses when participants were exposed to extreme height experience [13].

### 1.1. Improvisation and Social Rejection

Theatre-based practices, such as improvisation [14,15], frequently mimic reality, and parallel everyday social interactions. Improvisation is commonly understood as doing something ‘in the moment’, without a script or step-by-step preparation. One key idea we have adopted from this requirement is the concept of ‘knowing how to act in the moment’. In the teacher education context, this translates as how student teachers could be prepared to know how to deal with the unexpected in their future profession as teachers [16]. The current field of improvisation embraces a wide variety of on-stage forms, from theatre sports competitions to full-length plays, as well as applied forms, which utilise the improvisational skill set as a tool to benefit personal growth, education, or business. For instance, applied improvisation provides a model of reciprocal communication, and enables the step-by-step detection of how interaction is constructed (e.g., [17]).

Improvisation represents a concept of experiential learning. To learn experientially means not only engaging in doing something, but also reflecting on the experience of doing to increase knowledge on a topic [18]. Improvisation in education serves to create an interactive and positive learning environment in which participants’ construction of knowledge and learning takes place through functional and interactive social relationships. By alternately assuming a role and behaving as themselves, learners acquire operating experiences, and create new knowledge of the phenomena being reviewed [18]. Gallagher [19] finds the work of Keith Johnstone, an influential pioneer of modern improvisation and originally a classroom teacher himself, an effective model for working with new teachers who are keen to think more deeply about how improvised games might associate with their work in classrooms. For instance, the improvisation course mentioned at the beginning of this article serves as an example of this kind of thinking. As a part of a larger research project, we previously investigated the effects of improvisation training on student teachers’ social interaction competence [20,21]. Here, we focus specifically on the same student teachers’ psychophysiological reactivity during improvisation exercises.

In this study, we focus on improvisation training aimed at recognising rejective behaviour (negating any given idea verbally or nonverbally), since rejective behaviour prevents the improvisational scene from developing further and inhibits collaboration between partners. Improvisation is based on the principle of accepting any idea, referred to as ‘offers’ in improvisation. Offers are not evaluated as good or bad, but improvisers unconditionally accept any verbal or physical offer as a part of the scene (‘yes’) and add to it a new spontaneous association (‘and’). This is known as the basic ‘yes and’ rule of improvisation [14,15,22].

*‘I think we are finally ready to adopt a pet. What do you think?’*

*‘Yes! And it should definitely be a cat. They are so adorable and also easy to take care of.’*

*‘Yes, and why not have two as well, so they can keep each other company while we are at work.’*

Negating or rejecting is the opposite of accepting. Beyond total rejections—that is, refusing the partner’s offer completely—more subtle rejections are identified in improvisation as well. For instance, the general expression ‘but’ devalues and partially negates the meaning of what has been said previously, even when beginning with a positive ‘yes’: *‘Yes, your idea for adopting a cat is wonderful, but what if I am allergic to cats?’* In this study, we focused on three subtle rejections improvisation trains individuals to recognise: (1) devaluing (‘yes, but’); (2) interrupting the conversation partner; and (3) nonverbal rejection, such as showing signs of boredom, frowning, and avoiding eye contact among others while someone is speaking [23].

Although the present study contrasts real-life and fictional contexts, we do not imply that pursuing fictional experiences in an educational context should equate with real-world experiences in meeting learning objectives. Equivalent experiences might even become detrimental when exploring challenging topics, such as bullying at school or at work. Therefore, when simulating challenging social encounters, including social rejections, a psychologically safe environment is particularly important [24,25,26,27]. In improvisation, as in theatre-based practices in general, psychological safety is pursued through the use of fiction, such as through role-playing or distancing the challenging topic by using a fictional time or place. Cognitive distance (‘I know this is not real’) serves as a necessary and protective shield [6], allowing for safe emotional engagement in a controlled environment. Though the environment is fictional, holistic engagement [28,29,30,31] and experiential learning may emerge.

### 1.2. Biosignals of Social Rejection

A large body of research documents the behavioural, neural, and physiological responses generated by social rejections (i.e., situations that threaten or reject an individual’s social value) [32,33,34,35,36]. Social rejection threatens people’s fundamental need to belong, and may elicit negative mood, emotional distress, and reduced feelings of belonging, self-esteem, and control [32,34]. Furthermore, numerous findings indicate that social and physical pain share a partly common neuroanatomical basis in the human brain [8,34,37]. From an evolutionary perspective, a common neuronal circuitry appears adaptive, since social exclusion might have been a major threat to personal safety and survival [9]. Therefore, a social-evaluative threat triggers a coordinated psychophysiological and behavioural response to prevent social exclusion or the loss of one’s social status [38,39].

When exposed to a stressor, the autonomic nervous system (ANS) is triggered: specifically, the sympathetic nervous system is activated, and the parasympathetic nervous system is suppressed [40,41]. This stress response can be measured from the peripheral nervous system by measuring HR and electrodermal activity (EDA), commonly known as skin conductance [42,43,44,45]. HR acceleration is associated with a stress condition, such as a perceived social threat and social rejection [32,46]. However, other findings indicate that the HR slows for a few seconds in response to an unexpected social rejection [8,36,47]. This transient HR decelerating effect is thought to facilitate the processing of information in order to prepare for action and for adaptive performance during a socially threatening situation. In addition, a freezing response, representing a more sustained HR deceleration accompanied by behavioural immobility and increased muscle tonus for as long as the threat situation prevails, has been associated with a social threat [48,49,50,51]. Both a transient HR decelerating effect and a prolonged freezing response have been proposed as reflecting the initial, passive defence response to a social threat, such as social rejection [36,47,51].

Electrodermal activity, or a change in skin conductance, is a sensitive index of autonomic arousal under social stress (e.g., palms sweating during a stressful situation) (e.g., [9,52,53]). In the human body, eccrine sweat glands, found primarily on the palm side of the hands and the soles of the feet, are linked to the sympathetic branch of the ANS. Activation of these sweat glands can be measured as an increased level of electrical conductivity on the skin [54].

Physiological arousal may be detected using HR or EDA data, but these parameters do not differentiate the valence of the arousal (as positive or negative). Emotional specificity might be obtained by measuring the facial muscle activity using electromyography (EMG) [55]. Increased activity in the *zygomaticus major*—the facial muscle generating a smile—is associated with positive emotions, whereas the activity of the *corrugator supercilii*—the ‘frowning’ muscle, which pulls the eyebrows downwards and together—is associated with negative reactions [56,57]. This minuscule muscle activity can be detected even if a person shows no outward facial expression nor reports any conscious emotions [58]. Because the participants in our study reported their rejection-related responses following a dyadic interaction instead of pausing to report their emotions following each rejection, continuous and time-sensitive facial EMG data were essential to measuring the rejection-related emotional valence. However, the interpretation of facial EMG poses challenges and requires a thorough understanding of the context. For example, during social interactions, facial expressions might index the learned, socially acceptable emotion expression more than the inner emotional state or attentional processes, such as increased *corrugator supercilii* activity during concentration [59].

Finally, electroencephalography (EEG) is a non-invasive means of measuring electrical brain activity during social interactions. Specifically, the frontal EEG alpha asymmetry (EEG asymmetry) might be of interest for research on drama education, since the hemispheric differences in the frontal cortical activity associate with a motivation to approach or withdraw [60,61]. Studies across several stimulus modalities and age groups demonstrate that high levels of relative left-sided frontal activity associate with approach-related emotions, and high levels of relative right-sided frontal activity to negative, withdrawal-related emotions [62,63,64,65]. For example, Schmidt and Trainor [63] studied a similar phenomenon in the context of music, finding that regional EEG activity differentiated emotions along the dimensions of valence. Joyful and happy musical excerpts elicited greater left-sided EEG asymmetry, whereas fearful and sad musical excerpts elicited greater right-sided EEG asymmetry. Thus, EEG asymmetry might detect fleeting, unconscious responses for even the most subtle social rejections that do not reach the conscious mind, but which may nevertheless impact our behaviour [37]. In addition, Hari and Kujala [37] extended our knowledge regarding the neural underpinnings of social interaction for teaching, training, and therapy, as well as for understanding social conflicts and the effects of dyadic interactions.

### 1.3. Study Aim

How similar or different are real-life and fiction-induced social rejections as measured by psychophysiological reactivity? Answering this question might yield new insights into, and implications for, not only improvisation training, but theatre-based practices in general, given their use for educational purposes in various professional training programmes (e.g., teacher education) where social interaction skills are necessary. To this end, this study manipulated the fictionality of the context to investigate the modulation of psychophysiological responses to social rejections.

To do so, we measured participants’ HR, skin conductance, facial muscle activity, and electrocortical activity during an interview (real-life context) and improvisation exercises (fictional context). Both contexts involved three types of subtle social rejections: devaluing; interrupting; and nonverbal rejection. Rejections were unexpected during the interview, but were expected during the improvisation exercises.

In general, we hypothesised higher levels of physiological arousal (an accelerated HR and an elevated EDA) and negative valence (increased *corrugator supercilii* activity along with decreased activity in the *zygomaticus major*, and relative right-sided EEG asymmetry) as responses to social rejections compared to baseline measurements. We also hypothesised a stronger transient HR decelerating effect in the real-life relative to the fictional context, based on previous findings associated with unexpected social rejections. Otherwise, we adopted an exploratory study approach, given the ambiguous findings related to psychophysiological reactivity to the manipulation of real-life versus fictional stimuli.

## 2. Materials and Methods

### 2.1. Participants

In total, 39 healthy, right-handed, non-smoking undergraduate students at the University of Helsinki in Finland (33 women, 5 men, and 1 other) ranging in age from 20 to 40 years (M = 27.1, SD = 6.5) self-registered to the study via an automated system, and received course credit for study participation. The participants included 38 student teachers, 1 of whom studied a different, albeit related discipline (for further details on participants, see [20]). No participants reported present or past neurological disorders, the use of psychiatric medication, or a current illness. Participants had either no or negligible prior experience with improvisation. In addition, due to a poor signal quality and missing data, three participants were excluded from the further analysis of electrocardiography (ECG), EEG, and facial EMG data, and four participants were excluded from the EDA analysis.

The sample size (max = 20) was determined by the requirements of participatory teaching during the intervention, which was a part of this research project, aimed at enabling all participants to engage in active, experiential learning (intervention group *N* = 19, wait-listed control group *N* = 20). The statistical power achieved with *N* = 20 would, in general, allow for the detection of large effects as approximated by Cohen’s *d* at 1 − β = 0.80 when carrying out directional hypothesis testing [66]. Participants were informed about the study procedure, and provided their written consent to participate. Ethical approval for the study was obtained from the University of Helsinki Ethical Review Board for the Humanities and Social and Behavioural Sciences (statement 25/2017).

### 2.2. Design and Procedure

**Figure 1:**
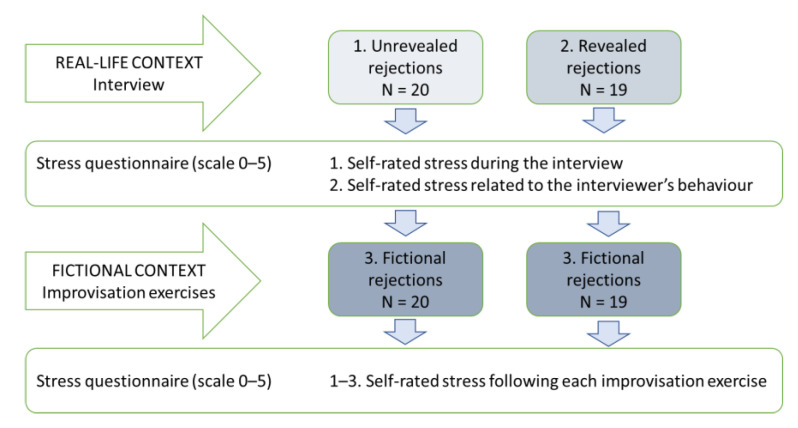
Study design.

The experimental design (Figure 1) contained three levels of awareness of fiction: (1) *unrevealed* rejections during the interview (that is, not aware of the fiction); (2) *revealed* rejections during the interview (partially aware of the fiction); and (3) *fictional* rejections during the improvisation exercises (fully aware of the fiction). However, each participant only experienced two levels of fiction: within an interview (unrevealed or revealed); and within fictional improvisation exercises. Each rejection type was repeated three to four times during the interviews, and three to five times during the improvisation exercises. The order of rejection exercises was counterbalanced across participants, as were the rejections during the interview, in an attempt to prevent an order effect. The contents of the rejections varied because the topics were related to participants’ personal experiences during the interview, and the participant was asked to create topics during the improvisation exercises.

Before the experiment, all participants completed an online demographic survey and two self-report questionnaires (interpersonal confidence and self-esteem), which were previously summarised [20]. All sessions began between 14:00 and 16:00 in order to control for circadian oscillations in physiological measures, and lasted for approximately 2.5 h. Participants were asked not to exercise strenuously 3 h or consume caffeinated beverages 2 h before the session. Upon arriving at the laboratory, participants were introduced to the laboratory facilities, provided with a general outline of the study, and asked to sign a written consent form. Then, electrodes were attached. The experiment began by recording two baseline conditions and the Trier social stress test (TSST) condition [67], the results of which appear elsewhere [21].

The study conditions reported here (interview as a real-life situation versus improvisation exercises as a fictional context) commenced after the TSST. Participants sat for a 10 min interview, which involved general questions about being a university student. The interview was described as representing a two-way interaction compared to the TSST tasks, which included a one-way interaction situation (a speech and a mental arithmetic task in front of a jury). Participants were unaware that the interviewer was an actor trained to include subtle social rejections during the interview. The participant was seated 2 m from the actor, behind a desk. Participants were randomly assigned to two groups: (1) unrevealed rejections group (*N* = 20); and (2) revealed rejections group (*N* = 19), in which participants were informed that the interviewer would act impolitely, but they were not told how. Following each question, the actor subtly rejected the participant by (1) devaluing, (2) interrupting, or (3) behaving nonverbally in a negative manner. Following the interview, the setup was revealed, the actor was dismissed, and participants reported their subjective psychological stress related to the interview.

Next, a researcher (first author) specialised in theatre improvisation conducted an improvisation exercise session, which lasted approximately 25 min. The session was comprised of two 2.5 min improvisation exercises, including a warm-up (word-by-word exercise), accepting (‘yes, and’), and total rejection exercises, as well as exercises of the same subtle rejections as those experienced during the interview. Before the rejection exercises, the researcher explained which rejection would be used. Here, we focus on the subtle rejection exercises as the other exercises fall beyond our scope and will be reported elsewhere. Participants reported their subjective psychological stress following each exercise. The interview and improvisation exercises were recorded with a high-resolution video camera (Logitech^®^ Webcam Pro 9000, Logitech International S.A., Lausanne, Switzerland). The video recordings were used to mark the beginning and end of rejections in relation to the physiological data. Each task was preceded by a silent 30 s waiting period with the physiological measurement and video recording already in progress. Synchronisation between the video recording and data was achieved by playing a short sine-wave sound after each waiting period.

### 2.3. Measurements

#### 2.3.1. Psychophysiological Data Collection

We used the LiveAmp wireless amplifier (Brain Products GmbH, Gilching, Germany) to record all physiological measurements with the sample rate set to 500 Hz. The EEG was recorded according to the International 10–20 system [68] using a standard 32-channel electrode cap (EASYCAP GmbH, Herrsching, Germany) from 26 electrode sites. Two sites were used to record the bipolar ECG, and four sites for the facial EMG. Recordings were completed using the actiCAP active electrodes with the ground electrode at location Fpz and the reference electrode at FCz. All electrode–skin impedances were ≤25 kOhm. The ECG electrodes were positioned at approximately 2 cm below the right and left collar bones. For EDA assessment, Ag/AgCl electrodes were placed on the first phalanx of the index and middle fingers of the non-dominant hand [69]. To record the facial muscle activity of the *corrugator supercilii* and *zygomaticus major*, bipolar electrode pairs were placed on the left side of the face according to international guidelines [70].

#### 2.3.2. Subjective Data Collection

Following the interview, participants reported (1) their self-rated stress during the interview, and (2) their self-rated stress related to the interviewer’s behaviour, using a Likert scale from 0 to 5 (0 = not at all stressed to 5 = extremely stressed). Following each improvisation exercise, participants reported (3) their self-rated stress during the exercise using the same scale.

### 2.4. Offline Physiological DATA Analysis

The silent 30 s waiting period preceding the interview was used as the baseline for HR, facial EMG, and EEG asymmetry (we excluded data from the first and last 10 s for HR, and from the first and last 5 s for facial EMG and EEG, due to a possible physiological reaction caused by the event signal). Epochs of 5 s (beginning with the rejective statement or the onset of a nonverbal rejection) were analysed. The rejection onsets were marked in relation to the physiological data based on the video recording using the ELAN 5.1 software (The Language Archive, Max Planck Institute for Psycholinguistics, Nijmegen, The Netherlands). The biosignal analyses were conducted using Matlab R2018a (Mathworks Inc., Natick, MA, USA) and EEGLAB 14.1.1b [71] unless otherwise stated.

The ECG signals were digitally filtered (1–30 Hz, FIR). The R peaks were extracted from the ECG signals using the Pan–Tompkins algorithm [72] implemented in Matlab. The RR intervals were visually inspected for large artefacts, and corrected (mean of the previous and next valid values) if the extreme values represented outliers.

For the HR deceleration analysis, HR time series from the 6 s preceding a rejection onset to 6 s following a rejection onset were segmented into 2 s intervals. Mean values (in beats per minute) for these six 2 s intervals were analysed. However, the HR deceleration effect was analysed for devaluing alone, since during interruption and nonverbal rejection, the more robust effect of turn-taking confounded the effect of the rejection. The interruption onset was simultaneous with the partner beginning to speak, and the onset of the nonverbal rejection was simultaneous with the participant beginning to speak. Turn-taking during devaluing occurred before the onset of a rejection, as the conversation partner first agreed with the participant, and then proceeded to make a devaluing statement starting with ‘but’ (marked as the onset of a rejection).

The EDA signals were low-pass filtered with a cut-off frequency of 15 Hz (FIR), and visually inspected for large artefacts (an amplitude change > 10 µS/s and clearly nonphysiological, due to movement, for instance). In the case of such artefacts, the signal was interpolated within the relevant time window using third-order polynomial fitting for 1.37% of the epochs. The EDA signals were epoched according to the rejections, and the mean of the signal was calculated for each epoch. For the EDA, data from the first second after a rejection onset was used as the local baseline, and data within 2 to 5 s were analysed for a rejection response. The extraction of the skin conductance responses (SCRs) was completed using an algorithm written by Robert Schleicher [73]. SimpleEDA algorithm detects spontaneous fluctuations in a signal by thresholding the slope speed (first derivative). The threshold parameter for the minimum slope speed was 0.5 nS/s, and 700 ms was marked as the minimum interval between two consecutive SCRs. The calculated properties consisted of the number of SCRs per minute, the mean amplitude of SCRs (amplitude at maximum minus amplitude at the base of the signal, where the slope was initially above the threshold), and the mean slope speed (rising edge) of the SCRs.

The facial EMG signals were filtered (30–130 Hz, FIR) and converted to bipolar measurement data by subtracting the channel data (lower minus upper for *zygomaticus major*, central minus distal for *corrugator supercilii*) from each other. The resulting signals were rectified and epoched according to rejections. The mean values from each 5 s epoch were calculated.

The first part of the offline EEG analysis was performed using the BESA software, version 7.0 (BESA GmbH, Gräfelfing, Germany), and comprised low-pass filtering at 0.5 Hz (forward phase, slope 6 dB/octave), high-pass filtering at 100 Hz (zero phase, slope 24 dB/octave), automatic and manual eye-blink correction using PCA, and the visual inspection of data. The criteria for channel interpolation were a disproportionate amplitude drift or a transient artefact (caused by sweating or movement) in the signal, and an atypical EEG frequency spectrum (i.e., strong 50 Hz mains hum). However, the Cz, F3, and F4 channels required no interpolation. Continuous EEG signals were exported from BESA to Matlab for further processing. The spectopo function of EEGLAB was used to calculate the power spectral density at an alpha band (8–12 Hz; [74,75]) for the re-referenced (Cz) and epoched signals. A window size of 500 samples, an overlap of 50%, and zero padding for 1024 samples were used, resulting in a frequency resolution of 0.49 Hz. Since the EEG alpha power is inversely related to the measurement of the cortical activity [76], lower levels of the alpha power indicate higher levels of cognitive activity. Asymmetry values were calculated by subtracting the log-transformed power spectral density at the alpha band of the left-electrode site from that of the right site (F4–F3), where negative scores imply a relatively stronger right-sided activation [77]. The mean (whole epoch) and maximum peak of the power spectral density values were analysed within the alpha frequency band.

### 2.5. Statistical Approach

Statistical analyses were performed using IBM’s SPSS Statistics, version 27.0. The manipulation check relied on repeated-measures analysis of variance (RM-ANOVA) to test whether the psychophysiological rejection responses differed from the baseline scores (planned contrasts).

The difference in self-reported stress during the unrevealed and revealed interview was analysed using the Mann–Whitney U-Test due to the non-normal distribution of the data. The effect of the rejection type for the self-reported stress during the improvisation exercises was tested using RM-ANOVA with REJECTION (devaluing, interruption, and nonverbal) as a within-subject factor.

The change scores for the rejection-related reactivity were calculated by subtracting the baseline from the rejection response values. All subsequent statistical tests were conducted using the change scores. Each physiological parameter (HR, EDA, facial EMG, and EEG) was analysed separately.

Correlation analyses were calculated to examine the association between the psychophysiological reactivity for real-life and fictional rejections for each rejection type separately. The primary research question regarding the differences in the psychophysiological reactivity between real-life and fictional rejections was tested using ANOVA for mixed measures with CONTEXT (real-life versus fictional) as a within-subject factor, and GROUP (unrevealed versus revealed interview) as a between-subject factor. A separate, similar ANOVA was conducted to determine the reactivity across all rejections, where the three rejections (devaluing, interruption, and nonverbal) were averaged as one variable (TOTAL). The results from both ANOVAs are presented in the same figures.

To analyse the HR deceleration effect, the mean for the six 2 s intervals were subtracted from each interval, producing a more time-sensitive index for a phasic HR change. A repeated-measures ANOVA for mixed measurements was performed for these change scores using TIME (six intervals) and CONTEXT (real-life versus fictional) as within-subject factors, and GROUP (unrevealed versus revealed interview) as a between-subject factor. Paired-samples t-tests were calculated as follow-up tests.

We set the alpha level to 0.05 for all statistical analyses. To counteract the increased probability of a Type 1 error (false-positive) in multiple comparisons, the false discovery rate (FDR) procedure [78] was performed for all post hoc comparisons across the study. We determined an a priori threshold of *q* < 0.1 to retain the balance between false positive and missed findings (false-negatives, or Type II errors). In the case of a violation of the sphericity assumption, we used the Huynh–Feldt adjustment. Estimates of the effect size are reported using the partial eta squared (η_p_^2^).

## 3. Results

### 3.1. Manipulation Check

An RM-ANOVA for TIME (baseline, rejection responses during the interview, and improvisation exercises) was performed for HR, facial EMG, and EEG asymmetry to examine whether rejection responses differed from baseline (a silent waiting period before the interview). For EDA, an RM-ANOVA for TIME (local baseline and rejection response) was calculated to determine the difference between rejection responses and local baseline (the first second following the onset of a rejection). Baseline values and rejection responses appear in Table A1 and Table A2 (Appendix A).

Results revealed significant main effects of time for HR (*F*(8, 280) = 9.680; *p* < 0.001; η_p_^2^ = 0.217), *zygomaticus major* (*F*(8, 280) = 14.128; *p* < 0.001; η_p_^2^ = 0.288), *corrugator supercilii* (*F*(8, 280) = 2.416; *p* = 0.015; η_p_^2^ = 0.065), EEG asymmetry mean (*F*(6, 210) = 2.633; *p* = 0.018; η_p_^2^ = 0.070); and EEG asymmetry peak (*F*(4.925, 172.385) = 2.363; *p* = 0.043; η_p_^2^ = 0.063).

The RM-ANOVA planned contrasts (Table 1) revealed that HR decelerated relative to baseline under all conditions excluding real-life and fictional interruptions. Rejections elevated the *zygomaticus major* activity under all conditions relative to baseline. *Corrugator supercilii* activity increased during real-life and fictional interruptions, as well as during real-life nonverbal rejections relative to baseline. The EEG asymmetry mean and peak were lower during real-life and fictional nonverbal rejections relative to baseline.

For the mean EDA, we observed a significant difference between the local baseline and rejection response during fictional devaluing (*F*(1, 35) = 7.793; *p* = 0.008; all *p*-values FDR corrected at *q* < 0.1; η_p_^2^ = 0.182), but not during other rejections (*p* for all > 0.05). For the SCR, the local baseline differed from the rejection responses during all rejections (*p* < 0.05 for all, Table 1). The RM-ANOVA planned contrasts revealed that the mean EDA was elevated during the fictional devaluing, and SCR was elevated during all rejections relative to baseline. Table 2 summarises the frequencies of the rejection types.

### 3.2. Correlations between Real-Life and Fictional Social Rejections

Table 3 presents the Spearman’s rank correlations between real-life and fictional social rejections. During all three rejection types, HR showed a strong, positive association, *zygomaticus major* showed a modest positive association, and *corrugator supercilii* showed a modest to strong positive association between real and fictional rejections. Both the mean and peak EEG asymmetry showed a modest, positive association during devaluing, and the mean EEG asymmetry revealed a modest, positive association during a nonverbal rejection. Skin conductance parameters showed no associations during the three rejection types. When we combined the rejection types, however, the mean EDA showed a nearly significant, positive association (*p* = 0.057) between real-life and fictional rejections. All other physiological parameters showed positive associations between combined real-life and fictional rejections.

### 3.3. Self-Reported Stress

The Mann–Whitney U-test identified no differences in self-reported stress between unrevealed (*Md* = 1.50) and revealed (*Md* = 2.00) interviews (*U* = 178.000, *Z* = −0.355, *p* = 0.749), nor between self-reported stress related to the interviewer’s behaviour during unrevealed (*Md* = 2.00) and revealed (*Md* = 1.00) interviews (*U* = 132.500, *Z* = −1.684, *p* = 0.107).

**Figure 2:**
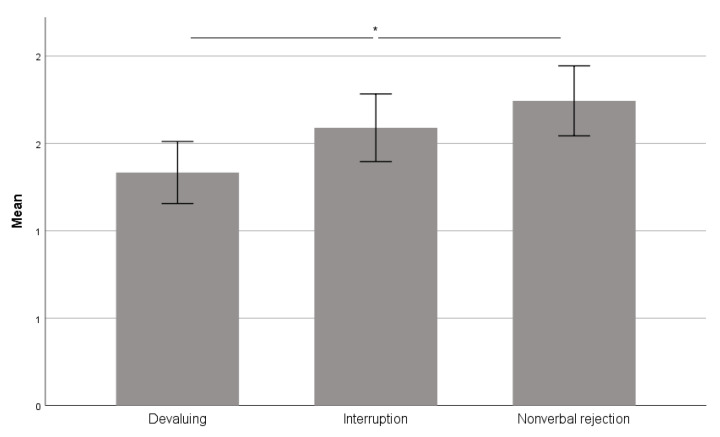
Self-reported stress during improvisation exercises. Scale 0 to 5 (0 = not stressed at all, 5 = extremely stressed). Error bars: +/−1 SE (* *p* < 0.05; FDR < 0.1).

The RM-ANOVA revealed a main effect for REJECTION (devaluing, interruption, and nonverbal) in the self-reported stress during the improvisation exercises (*F*(2, 76) = 4.777; *p* = 0.011; η_p_^2^ = 0.112). Pairwise comparisons revealed that nonverbal rejection was evaluated as more stressful than devaluing (Figure 2; *p* = 0.010).

### 3.4. Physiological Differences between Real-Life and Fictional Social Rejections

We performed mixed-measure ANOVAs to determine whether the rejection reactivity differed depending upon the fictionality of the CONTEXT (real-life versus fictional) and the GROUP (unrevealed versus revealed interview).

We observed no main effects for GROUP (unrevealed versus revealed interview; *p* > 0.05 for all), indicating no between-group differences existed based on the rejection reactivity regarding the type of interview. Furthermore, we detected no interactions between CONTEXT and GROUP (*p* > 0.05 for all), indicating that the type of interview did not influence the rejection reactivity during real-life or fictional rejection. Therefore, the GROUP factor was excluded from the statistical models, and we performed the repeated-measure ANOVAs using CONTEXT (real-life versus fictional) as the within-subject factor.

The RM-ANOVA results revealed significant main effects for CONTEXT (real-life versus fictional) for HR, mean EDA, SCR, and *zygomaticus major* activity (Table 4), indicating a differential rejection reactivity during the interview (real-life) and improvisation exercises (fictional). Yet, we observed no main effects for *corrugator supercilii* activity nor EEG asymmetry (*p* > 0.05 for all).

When the rejection reactivity was examined across all rejections (TOTAL), the RM-ANOVA results revealed significant main effects for CONTEXT (real-life versus fictional) for HR and SCR (Table 5).

**Figure 3:**
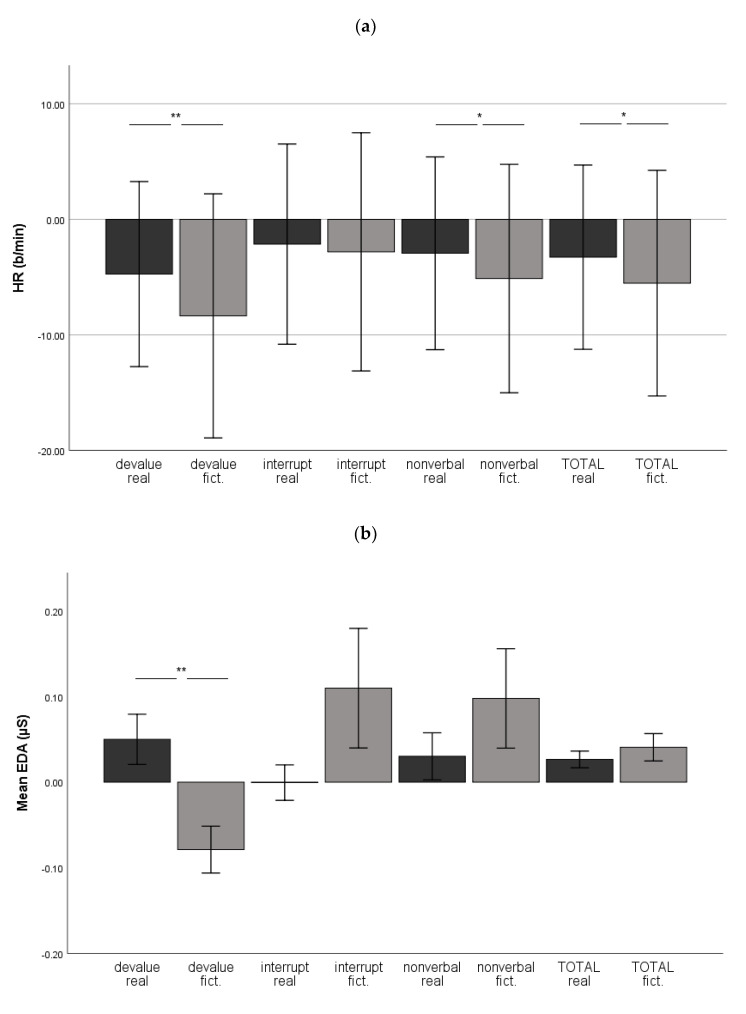

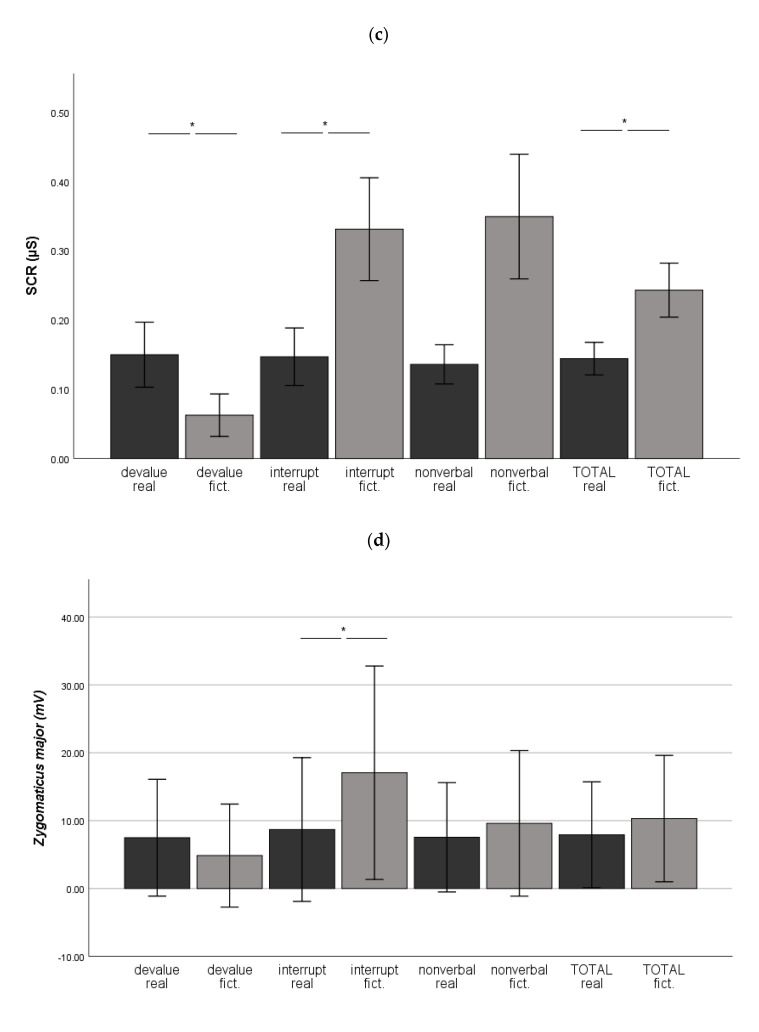

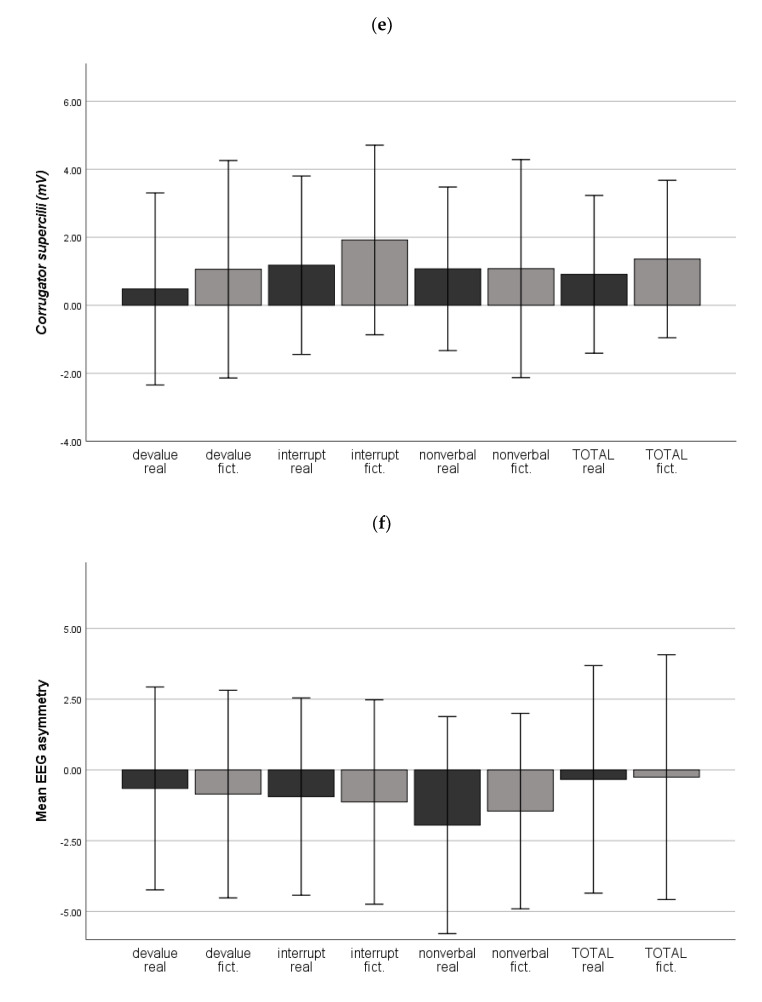

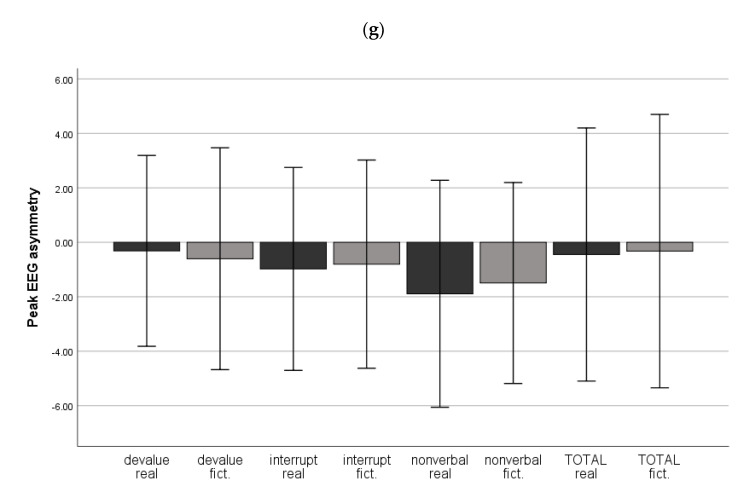
Physiological responses during real-life (real) and fictional (fict.) social rejections. Values represent the change scores (baseline subtracted). (**a**) Heart rate (HR), (**b**) mean EDA, (**c**) skin conductance response (SCR), (**d**) *zygomaticus major*, (**e**) *corrugator supercilii*, (**f**) mean EEG asymmetry, and (**g**) peak EEG asymmetry. Error bars: +/−1 SE (* *p* < 0.05, ** *p* ≤ 0.001, FDR < 0.1).

Post hoc tests using the FDR correction indicated that HR was significantly lower during fictional devaluing, nonverbal rejection, and when rejections were combined relative to real-life rejections (Figure 3a). In addition, the mean EDA was significantly lower in fictional devaluing relative to real-life devaluing (Figure 3b). Moreover, SCR was significantly lower during fictional devaluing, and higher during fictional interruption, as well as when rejections were combined relative to real-life rejections (Figure 3c). *Zygomaticus major* activity was significantly higher during a fictional interruption relative to a real-life interruption (Figure 3d). However, *corrugator supercilii* activity (Figure 3e) and EEG asymmetry (mean in Figure 3f; peak in Figure 3g) failed to differentiate between real-life and fictional rejections.

#### HR Deceleration Effect

**Figure 4:**
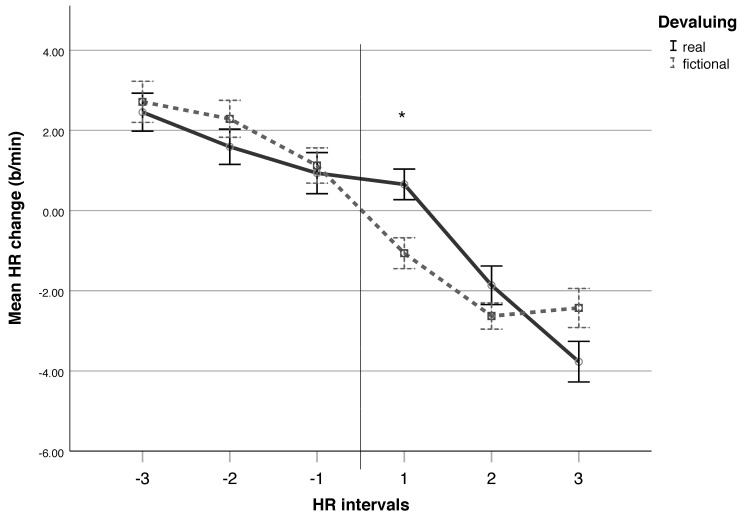
Phasic HR response to social rejection (devaluing). Here, 2 s HR intervals from 6 s before the onset of a rejection (−3, −2, and −1) to 6 s after the onset of a rejection (1, 2 and 3). The time of the rejection onset is presented as the vertical line. *N* = 38. Error bars: +/−1 SE (* *p* < 0.05, FDR < 0.1).

We analysed six 2 s intervals from 6 s before the onset of a rejection (intervals of −3, −2, and −1) to 6 s following the onset of a rejection (intervals of 1, 2 and 3). ANOVA using TIME (intervals −3 to 3) and CONTEXT (real-life versus fictional) as within-subject factors, and GROUP (unrevealed versus revealed interview) as a between-subject factor identified a main effect of TIME (*F*(5, 180) = 43.097; *p* < 0.001; η_p_^2^ = 0.545) and a TIME*CONTEXT interaction (*F*(5, 180) = 2.344; *p* = 0.043; η_p_^2^ = 0.061). Pairwise comparisons revealed that all post-rejection intervals (1, 2, and 3) differed from the pre-rejection intervals (−3, −2, and −1; *p* < 0.05 for all), indicating an HR deceleration effect. Subsequent paired-samples t-tests identified a difference between real-life and fictional rejections at the first post-rejection interval (*t*(37) = 3.245; *p* = 0.002), indicating a larger HR deceleration during fictional devaluing (Figure 4).

## 4. Discussion

Previous studies employed digital games, visual stimuli, and audio recordings to investigate the effects of social rejections on behavioural and psychophysiological responses [8,32,35,36,47,79]. The rejective stimuli have been quite robust, ranging from laughter and insults [35], to ostracism [8,9,10]. Here, we studied rejections so mild and subtle that they often remain unnoticed by the person rejecting—perhaps unintentionally, such as by interrupting due to enthusiasm—or even by the person rejected. Nevertheless, these subtle social rejections may distract from mutual understandings, and restrict collaboration at a subconscious level. In terms of the paradox of fiction, the effects of real-life versus fictional stimuli have been explored using photographs and video clips [5,6,11,30]. We used a theatre-based improvisation method to recreate dyadic interactions, and compared student teachers’ psychophysiological responses to fictional social rejections (devaluing, interrupting, and nonverbal rejection) to real-life rejections. Here, we combined these lines of inquiry, and extended them further by using naturally unfolding face-to-face rejections as the experimental stimuli. Furthermore, instead of a dual real life–fictional contrast, we manipulated the degree of fictionality by creating three levels of fiction. We analysed student teachers’ heart rates (HRs), skin conductance (mean EDA and SCRs), facial muscle activity (*zygomaticus major* and *corrugator supercilii*), and electrocortical activity (mean and peak EEG alpha asymmetry) during a 5 s time window beginning from the onset of rejections.

In the interview context, we manipulated the degree of fictionality by assigning participants to two randomised groups: (1) *unrevealed* rejections (that is, unaware of the fiction); and (2) *revealed* rejections, whereby participants knew that the interviewer would act impolitely, although not specifically in what manner (that is, partly aware of the fiction). Both self-reports and psychophysiological measurements failed to identify between-group differences, possibly indicating similar processing of rejections regardless of their fictional degree. However, it is also possible that the contrast was simply too weak to be detected by the measurements used in this study. Furthermore, perhaps the revealed impoliteness of the interviewer prompted a heightened detection of rejections and related physiological responses. Nonetheless, since we observed no differences, the two levels of the interview were combined into a single category (real-life context), which was compared with (3) *fictional* rejections during improvisation exercises (that is, fully aware of the fiction).

### 4.1. The Success of the Stimuli Manipulation

We succeeded in generating relatively mild and subtle social rejections as intended (self-reported stress M ≤ 1.74, Md ≤ 2.00 on a Likert scale from 0 to 5 for all conditions). Though rejections were evaluated as mild, they elicited rejection-related physiological arousal, indicated by the elevated levels of SCRs compared to baseline. Furthermore, the EEG asymmetry values were negative during all conditions, indicating that rejections evoked withdrawal-related behaviour as expected. *Corrugator supercilii* activity increased relative to baseline also as expected, but only during nonverbal rejections in the real-life context, and during interruptions in the real-life and fictional contexts.

However, contrary to our expectations, HR decreased across all conditions relative to baseline, measured during the silent waiting period before the real-life context, that is, during the interview. Participants might have been nervous whilst anticipating the interview, resulting in an elevated waiting-period HR. Another explanation might lie in the incomplete recovery from the preceding Trier social stress test (TSST) condition [21]. This explanation is supported by a study from Kudielka et al. [80], which revealed a gender effect for HR recovery, whereby women showed less HR recovery than men during a 5 min period following TSST. The majority of participants in our study were women (84.6%), and the time span between the end of TSST and the beginning of baseline recording reached a maximum of 5 min. Thus, the previous test condition might have inflated the baseline measurement, which should be taken into consideration when interpreting the HR results. For instance, we cannot infer that anticipating the interview was more arousing than social rejections during the study, as indicated by the negative rejection-related values (change scores where the baseline was subtracted from the rejection response).

### 4.2. The Relationship between Real-Life and Fictional Social Rejections

#### 4.2.1. Devaluing (‘Yes, but’)

The measures of HR and skin conductance indicated heightened cardiac and sympathetic arousal during the real-life interview relative to the fictional improvisation. However, the indicators of valence (facial EMG and EEG asymmetry) failed to distinguish between real-life and fictional contexts. A closer cardiac beat-by-beat analysis revealed that both contexts generated an HR deceleration effect, becoming more distinct during improvisation, where HR slowed immediately following the onset of a rejection, although during the interview, HR deceleration was observed 2 s later. This result was surprising, since we hypothesised a stronger deceleration response during the unexpected rejections during the interview compared to the expected, and even explicitly specified rejections during the improvisation exercises. A dissimilar speech tempo between the interviewer and researcher (a slower speech tempo of the interviewer) may explain this unexpected finding. However, this possibility remains speculative until verified by a closer analysis of speech, which lies beyond the scope of this study. Nevertheless, given that an HR deceleration effect serves as a direct indicator of the bodily experience of social rejection, our results suggest that both real-life and fictional devaluing generate transient rejection-related cardiac reactivity, regardless of the expectation or level of the pre-rejection HR.

#### 4.2.2. Interruption

*Zygomaticus major* activity and SCR were stronger during fictional versus real-life interruptions, whereas HR, mean EDA, *corrugator supercilii,* and EEG asymmetry failed to differ between real-life and fictional interruptions. Thus, the physiological arousal indicated by HR and mean EDA was similar in real-life and in fictional contexts, alongside the negative valence expression indicated by *corrugator supercilii* and the withdrawal-related motivation indicated by EEG asymmetry. This similarity appears plausible, because an interruption represents a complete and abrupt event in dyadic interactions, forcing a person to cease speaking, and to begin listening. The bodily change in activity is rather robust, regardless of the fictionality of the situation.

Because the activity of the *zygomaticus major* is generally associated with positively valenced emotions and appeared heightened in a fictional context, it is possible to infer that the improvised interruptions were more pleasant or amusing than real-life interruptions. However, the negatively valenced *corrugator supercilii* activity did not diminish, rendering our interpretation regarding pleasantness ambiguous. Ravaja et al. [81] found somewhat similar results in their game study, whereby several putatively negative game events prompted facial EMG responses of a positive valence. Here, *zygomaticus major* activity might also reflect emotion contagion (that is, a transfer of emotions between communicating persons), as the drama instructor often acted enthusiastically whilst interrupting, whereas the interaction during the interview was presumably more official. Thus, the interpretation of *zygomaticus major* activity during interruptions remains unclear. However, because the improvised interruptions were identified as more arousing than real interruptions using SCR, it is reasonable to interpret that, regardless of the valence, fictional interruptions generated more sympathetic arousal relative to real-life interruptions.

#### 4.2.3. Nonverbal Rejection

Facial EMG, EEG asymmetry, and skin conductance did not differ between real-life and fictional nonverbal rejections, indicating a similar rejection-related valence, withdrawal motivation, and sympathetic arousal, respectively. The single measure differing between real-life and fictional nonverbal rejections was HR, decelerating during the fictional context compared to the real-life context. Because the heart is controlled by both sympathetic and parasympathetic branches of the ANS, and no difference was observed in sympathetic activity, the parasympathetic activity might play a role [46] in lowering HR during fictional nonverbal rejections. The parasympathetic activity here may have reflected an emotional downplay associated with fictional stimuli (e.g., [5,30]).

#### 4.2.4. Combined Rejections

When all three rejection types were averaged, five physiological parameters did not distinguish between real-life and fictional rejections (mean EDA, *zygomaticus major*, *corrugator supercilii,* and mean and peak EEG asymmetry). The measures of HR and SCR differed between real-life and fictional contexts, instead yielding contradictory results, as an elevated HR in a real-life context suggested a greater arousal relative to a fictional context, and an elevated SCR in a fictional context suggested a greater arousal relative to a real-life context (for other differential HR and skin conductance results, see, e.g., [32,82]). However, one explanation is that the increased sympathetic arousal observed through a high SCR associated more with cognitive effort [83] than with an emotional arousal in the fictional context because participants performed improvisation exercises for the first time during the experiment.

#### 4.2.5. Correlations between Real-Life and Fictional Rejections

Given the lack of research on psychophysiological reactivity association during real-life and fictional social rejections, our study approach during the correlation analyses was exploratory. We observed a modest to strong positive association between real-life and fictional rejections for the HR, EMG, and EEG parameters. We observed no associations for the skin conductance parameters. When rejection types were combined, the physiological parameters showed a positive association between real-life and fictional rejections, excluding the mean EDA, which showed a positive tendency (*rho* = 0.315, *p* = 0.057). These associations might indicate comparable processing of real-life and fictional stimuli, discussed next within the framework of emotion regulation.

#### 4.2.6. The Impact of Personal Relevance on Processing Real-Life versus Fictional Stimuli

Previous research on the paradox of fiction indicates the existence of an emotional downplay from fictional stimuli, resulting in weaker responses relative to real-life stimuli. For instance, Mendelson and Papacharissi [6] suggest that our brains are prepared to use different strategies to understand the meaning of an event when the nature of the event (real-life versus fictional) is known beforehand. Moreover, Mocaiber et al. [5] propose that affective processing is context-dependent. Additionally, in their functional magnetic resonance imaging (fMRI) study, Abraham et al. [84,85] observed a fine distinction in the way conceptual information related to real persons versus the way fictional characters were represented in the brain. Abraham et al. [84] also introduced *self-relevance* as a possible moderating factor in the processing of real-life versus fictional content, suggesting that real entities are more personally relevant than their fictional counterparts. Similarly, Sperduti et al. [30] found a modulating effect from self-relevance in their study on emotional responses toward video clips labelled real or fictional. They suggested that, given knowledge of the fictional nature of the stimulus, implicit emotion regulation takes place, resulting in a weaker self-reported emotional response compared to real-life stimulus. However, fictional stimuli that elicit personal, self-relevant memories may counterbalance this effect, and prompt an emotional arousal.

This aspect of self-relevance might explain why we found no systematic attenuation of the psychophysiological arousal or valence related to the fictionality of the stimuli (e.g., fictional interruptions were more arousing than real-life counterparts, *corrugator supercilii* activity and EEG alpha asymmetry did not distinguish between real-life and fictional contexts). Previous studies used images and videos as stimuli, whereas we used face-to-face social rejections in a naturally unfolding dyadic interaction. Here, the situation required personal engagement with spontaneous ideas from the participants’ own imagination. It is possible that rejecting these self-relevant ideas might have counteracted the suggested fiction-related downplaying processes, and resulted in psychophysiological responses relatively comparable to those from real-life rejections. This explanation is highly interesting from the perspective of theatre-based practices in educational contexts, because they rely on the holistic action and the personal engagement of participants.

### 4.3. Implications of the Research

The findings of this study benefit the literature on social exclusion, because the study focuses on rather subtle rejections, which are much more common in real-life than the more artificial rejection manipulations that dominate the literature. These findings provide new information on dyadic social interactions, showing that even relatively mild and subtle social rejections prompt distinctive physiological reactions. Additionally, all rejections elicited a negative EEG asymmetry, associated with a behavioural withdrawal motivation. Therefore, becoming aware of and avoiding our own unnecessary subtle rejections is motivating, at least when pursuing positive social interactions. Specifically, awareness of one’s nonverbal behaviour appears essential, because participants identified nonverbal rejections as more stressful than devaluing, and nonverbal rejections generated the most similar responses when comparing real-life and fictional contexts.

The current study also provides new information to the field of education in demonstrating that realistic social situations can be explored and practiced experientially in a fictional context for learning purposes. Regardless of the fictionality of the context, genuine emotions and experiences may emerge, thereby serving experiential learning.

Furthermore, this study contributes to social neuroscience by suggesting how naturally unfolding social interactions can be achieved in an experimental design using improvisation. Improvisational face-to-face exercises can be performed while sitting still, and they possess a certain recurring shape, allowing the necessary repetition required in neuroscientific experimental designs. Moreover, one strictly defined issue of social interaction can be presented at a time, such as approval, rejection, or different levels of subtle rejections.

### 4.4. Limitations of the Research

In this study, the controllability of the stimuli was compromised. First, instead of presenting discrete and static stimuli (e.g., images or sounds) to passive participants, we used improvised social rejections during active, dyadic interactions. Second, instead of using an identical set of stimuli, each stimulus (rejection) was unique, given the diverse contents of the conversations spontaneously determined by participants. We hypothesised that if an effect related to a fictional degree or a rejection type exists independent from the conversational content, it should be identified through an adequate number of repetitions. However, this study would have benefitted from a larger number of rejections. In addition, perhaps the fiction- and rejection-related effects were covered by the overall stress caused by the test situation (that is, the interview, improvisation exercises for the first time, and attached psychophysiological measurement equipment).

In terms of the cardiac deceleration effect, we did not obtain a typical HR recovery following the rejection-related deceleration, given the relatively short duration of the post-stimulus time window. As we attempted to use a naturally unfolding conversation and improvised rejections, we were unable to control the duration of the conversational turn-taking. Using a longer post-stimulus time window would have reduced the amount of usable data (when the participant was silent and exposed to a devaluing rejection). Nevertheless, we could maintain a dynamic flow of dyadic interactions, where persons respond to each other’s spontaneous behaviour. Przyrembel et al. [86] stated that, in order to reflect real social interactions, an experimental design should allow for dynamic interplay, a virtually unlimited range of responses, living and uncontrolled partners, and emergent qualities. Theatre-based improvisation meets all four of these criteria for real social interactions. This trade-off seems worthwhile because the ecological validity of the stimuli appears to make a significant difference—that is, the complex and life-like stimuli seem to activate brains more, and differently, than discrete and static stimuli [87,88].

Furthermore, we did not counterbalance the order of the conditions: improvisations always followed the interview, which was unavoidable. Had the improvisation exercises preceded the interview, participants might have guessed the purpose of the interview, and the real-life nature of the interview would have suffered. Still, it is possible that participants doubted the authenticity of the interview assuming that it was another fictional test condition, possibly explaining the congruence between the physiological responses during the interview and the improvisation exercises. However, the interview was ego-involving and required processing of actual, personal experiences. Evidence suggests that ego-involving experimental protocols generate strong physiological reactions comparable to real-life experiences, such as the TSST [67], despite participants’ knowledge that the test was created for research purposes. Furthermore, half of the participants were naïve to the intention of the experiment regarding the physiological reactivity to social rejections. The other half of the participants, who were informed about the impoliteness of the reviewer (the revealed rejections group), and might have further suspected the authenticity of the setup, showed no differences in their physiological reactivity relative to the naïve group. Likewise, in a study by Linden et al. [89], awareness of the intention of the experiment did not alter the physiological and affective stress reactivity.

In addition, the lack of any counterbalancing raises questions related to the order effect and possible overall relaxed state of the participants. First, perhaps the experience of the interview influenced the subsequent fictional condition. Therefore, instead of the within-subject experimental design of this study, future research should employ a between-subject design—although masking the intention of the study might prove problematic, and interindividual variability should be controlled. Second, the overall relaxation of the participants towards the end of the experiment remains a valid concern, possibly explaining the decreased physiological effects in a fictional relative to a real-life context. However, we also observed higher levels of physiological arousal (SCR) and valence (*zygomaticus major*) in fictional relative to the preceding real-life condition. Therefore, any concern regarding the relaxation of participants as an explanatory factor appears unsupported.

### 4.5. Suggestions for Further Research

This study explored the psychophysiological effects of real-life and fictional social rejections within a 5 s post-stimulus time window. It would be highly interesting to explore the effect with more time-sensitive measures, such as event-related potentials (e.g., ERP, direct brain response to a specific event measured by EEG). Mocaiber et al. [5] studied picture-related ERPs, and found evidence for an implicit downplaying of the stimulus relevance in a fictional versus real-life context. This top-down control was observed within a post-stimulus time window of 300 to 600 ms. In this study, an ERP approach could not be used since the number of the event repetitions would not have been sufficient for ERP analysis. Extending this approach to naturalistic face-to-face interactions would be challenging, but possible, as demonstrated by Goregliad Fjaellingsdal et al. [90] in their ERP study of dyadic interactions. Furthermore, hyperscan techniques, where the brain activity of two or more persons are monitored simultaneously (see, e.g., [91]), would advance our understanding of the neural underpinnings of communicative processes related to social rejections. This approach might involve examining the emotional contagion or the synchronisation/desynchronisation of neural activity (e.g., [92]).

## 5. Conclusions

This study contributes to research on the paradox of fiction by comparing psychophysiological responses elicited by real-life and fictional social rejections to explore how awareness of fiction influences the experience of social rejection.

We found that psychophysiological responses in real-life and fictional contexts were associated with one another. The strength of the association varied depending on the biosignals, with the strongest association found for the heart rate. Furthermore, the rejection type modified the response differences between real-life and fictional contexts as measured by heart rate, skin conductance, and facial *zygomaticus major* activity. Facial *corrugator supercilii* activity and EEG asymmetry did not distinguish between real-life and fictional contexts during any of the social rejections. Overall, no systematic fiction-related attenuation of the psychophysiological arousal or valence was detected.

In conclusion, this study provides a method for integrating theatre-based improvisation with neuroscientific measurements during dyadic interactions, and shows that psychophysiological responses during improvisation exercises are relatively congruent with real-world responses, regardless of the cognitive awareness of fictionality. The results provide novel, biological support for the application of improvisation as a tool to train social interaction competence, for example, in professions where face-to-face interaction is required.

## Figures and Tables

**Table 1:** Differences between Baseline and Social Rejection Responses.

			Devaluing vs. Baseline			Interruption vs. Baseline			Nonverbal Rejection vs. Baseline		
	*N*	df	F	*p*	η_p_^2^	F	*p*	η_p_^2^	F	*p*	η_p_^2^
**Real-life context (interview)**											
Heart rate (HR)	36	(1,35)	13.594	0.001 **	0.280	2.509	0.122	0.067	4.335	0.045 *	0.110
Mean EDA	38	(1,37)	2.902	0.097	0.073	0.001	0.978	0.000	1.202	0.280	0.031
Skin conductance response (SCR)	38	(1,37)	10.131	0.003 *	0.215	12.406	0.001 **	0.251	22.751	<0.001 **	0.381
*Zygomaticus major*	36	(1,35)	25.509	<0.001 **	0.422	23.791	<0.001 **	0.405	28.141	<0.001 **	0.446
*Corrugator supercilii*	36	(1,35)	0.895	0.351	0.025	6.171	0.018 *	0.150	5.410	0.026 *	0.134
EEG asymmetry, mean	36	(1,35)	1.621	0.211	0.044	3.642	0.065	0.094	10.754	0.002 *	0.235
EEG asymmetry, peak	36	(1,35)	0.435	0.514	0.012	2.810	0.103	0.074	7.503	0.010 *	0.177
**Fictional context (improvisation exercises)**											
Heart rate (HR)	36	(1,35)	20.662	<0.001 **	0.371	2.685	0.110	0.071	9.131	0.005 *	0.207
Mean EDA	36	(1,35)	7.793	0.008 *	0.182	2.472	0.125	0.066	2.799	0.103	0.074
Skin conductance response (SCR)	36	(1,35)	4.183	0.048 *	0.107	19.908	<0.001 **	0.363	14.537	0.001 **	0.293
*Zygomaticus major*	36	(1,35)	15.109	<0.001 **	0.302	42.371	<0.001 **	0.548	27.320	<0.001 **	0.438
*Corrugator supercilii*	36	(1,35)	3.652	0.064	0.094	17.078	<0.001 **	0.328	3.131	0.086	0.082
EEG asymmetry, mean	36	(1,35)	2.660	0.112	0.071	3.541	0.068	0.092	7.366	0.010 *	0.174
EEG asymmetry, peak	36	(1,35)	0.708	0.406	0.020	1.116	0.298	0.031	5.589	0.024 *	0.138

Note: df, degrees of freedom; η_p_^2^, partial eta squared (effect size); EDA, electrodermal activity. Significant results are marked with **bold font**. * *p* < 0.05; FDR < 0.1. ** *p* ≤ 0.001; FDR < 0.1.

**Table 2:** Frequencies of REJECTION types.

	Devaluing	Interruption	Nonverbal Rejection	Total
Real context (interview)	151	163	122	436
Fictional context (improvisation drills)	128	116	198	442

**Table 3:** Spearman’s Rank Correlations for Physiological Responses Between Real-Life and Fictional Social Rejections.

	Real-Life vs. Fictional Devaluing			Real-Life vs. Fictional Interruption			Real-Life vs. Fictional Nonverbal Rejection			Real-Life vs. Fictional TOTAL Rejections		
	*rho*	*p*	*N*	*rho*	*p*	*N*	*rho*	*p*	*N*	*rho*	*p*	*N*
Heart rate (HR)	0.805 **	<0.001	38	0.718 **	<0.001	36	0.812 **	<0.001	38	0.843 **	<0.001	38
Mean EDA	−0.078	0.644	37	−0.062	0.725	35	−0.014	0.932	37	0.315	0.057	37
Skin conductance response (SCR)	−0.016	0.925	37	0.103	0.555	35	0.053	0.756	37	0.515 **	0.001	37
*Zygomaticus major*	0.551 **	<0.001	38	0.531 **	0.001	36	0.686 **	<0.001	38	0.617 **	<0.001	38
*Corrugator supercilii*	0.592 **	<0.001	38	0.495 **	0.002	36	0.733 **	<0.001	38	0.412 *	0.010	38
EEG asymmetry, mean	0.450 **	0.005	38	0.091	0.596	36	0.337 *	0.038	38	0.627 **	<0.001	38
EEG asymmetry, peak	0.468 **	0.003	38	0.158	0.358	36	0.294	0.073	38	0.677 **	<0.001	38

Note: rho, Spearman’s rho; TOTAL, mean of the combined rejection types (devaluing, interrupting, and nonverbal). Significant results are marked with **bold font**. * *p* < 0.05. ** *p* ≤ 0.001.

**Table 4:** Differences in Psychophysiological Reactivity between Real-Life and Fictional Social Rejections.

			Real-Life vs. Fictional Devaluing			Real-Life vs. Fictional Interruption			Real-Life vs. Fictional Nonverbal Rejection		
	*N*	df	F	*p*	η_p_^2^	F	*p*	η_p_^2^	F	*p*	η_p_^2^
Heart rate (HR)	36	(1,35)	11.552	0.002 *	0.248	0.182	0.672	0.005	5.579	0.024 *	0.137
Mean EDA	35	(1,34)	13.590	0.001 *	0.286	2.814	0.103	0.076	0.962	0.334	0.028
Skin conductance response (SCR)	35	(1,34)	6.148	0.018 *	0.153	4.945	0.033 *	0.127	3.795	0.060	0.100
*Zygomaticus major*	36	(1,35)	3.192	0.083	0.084	10.482	0.003 *	0.230	2.323	0.136	0.062
*Corrugator supercilii*	36	(1,35)	1.266	0.268	0.035	1.736	0.196	0.047	0.008	0.927	0.000
EEG asymmetry, mean	36	(1,35)	0.193	0.663	0.005	0.003	0.955	0.000	0.831	0.368	0.023
EEG asymmetry, peak	36	(1,35)	0.167	0.685	0.005	0.158	0.693	0.005	0.355	0.555	0.010

Note: df, degrees of freedom; η_p_^2^, partial eta squared (effect size); EDA, electrodermal activity. Significant results are marked with **bold font**. * *p* < 0.05; FDR < 0.1.

**Table 5:** Differences in Psychophysiological Reactivity for Combined Social Rejections between Real-Life and Fictional Contexts.

			Real-Life vs. Fictional TOTAL Rejections		
	*N*	df	F	*p*	η_p_^2^
Heart rate (HR)	38	(1,37)	7.556	0.009 *	0.170
Mean EDA	37	(1,36)	1.277	0.266	0.034
Skin conductance response (SCR)	37	(1,36)	10.693	0.002 *	0.229
*Zygomaticus major*	38	(1,37)	3.129	0.085	0.078
*Corrugator supercilii*	38	(1,37)	1.183	0.284	0.031
EEG asymmetry, mean	38	(1,37)	0.032	0.859	0.001
EEG asymmetry, peak	38	(1,37)	0.068	0.796	0.002

Note: df, degrees of freedom; η_p_^2^, partial eta squared (effect size); EDA, electrodermal activity; TOTAL, mean of the combined rejection types (devaluing, interruption, and nonverbal rejection). Significant results are marked with **bold font**. * *p* < 0.05; FDR < 0.1.

## Data Availability

The research data are confidential.

## Appendix A

**Table A1:** Baseline Values and Social Rejection Responses (HR, Facial EMG and EEG Asymmetry).

		Heart rate (HR)		*Zygomaticus major*		*Corrugator supercilii*		EEG Asymmetry, Mean		EEG Asymmetry, Peak	
		M	SD	M	SD	M	SD	M	SD	M	SD
	Baseline	83.44	14.22	6.81	4.90	6.42	4.74	−0.02	3.38	−0.24	3.50
Real	Devaluing	78.70	10.27	14.34	11.31	6.81	4.86	−0.65	2.66	−0.32	2.80
	Interruption	81.29	10.66	15.55	13.68	7.51	4.77	−0.94	3.09	−0.98	3.45
	Nonverbal rejection	80.50	10.24	14.40	9.48	7.41	5.62	−1.95	3.22	−1.89	3.41
	TOTAL	80.16	10.10	14.77	10.59	7.25	4.95	−1.18	2.46	−1.06	2.68
Fict.	Devaluing	75.09	8.12	11.71	9.20	7.39	6.03	−0.85	2.71	−0.60	3.13
	Interruption	80.52	9.01	24.17	16.98	8.44	5.11	−1.17	3.03	−0.84	3.32
	Nonverbal rejection	78.32	7.80	16.46	12.23	7.41	4.74	−1.46	2.64	−1.50	2.99
	TOTAL	77.92	7.78	17.16	11.07	7.70	4.91	−1.10	2.28	−0.94	2.62

Note: TOTAL, mean of combined rejection types (devaluing, interruption, and nonverbal).

**Table A2:** Baseline Values and Social Rejection Responses (EDA).

		Mean EDA Baseline (1 s)		Mean EDA Response (2–5 s)		SCR Baseline (1 s)		SCR Response (2–5 s)	
		M	SD	M	SD	M	SD	M	SD
Real	Devaluing	18.87	7.48	18.92	7.51	18.95	7.51	19.10	7.58
	Interruption	18.96	7.53	18.96	7.53	19.02	7.54	19.16	7.58
	Nonverbal rejection	18.89	7.46	18.92	7.46	18.96	7.47	19.09	7.50
	TOTAL	18.91	7.48	18.93	7.49	18.98	7.50	19.12	7.54
Fict.	Devaluing	21.50	10.24	21.42	10.27	21.61	10.29	21.67	10.39
	Interruption	21.90	10.83	22.01	10.79	22.05	10.87	22.38	10.99
	Nonverbal rejection	21.59	10.08	21.69	10.10	21.69	10.12	22.04	10.26
	TOTAL	21.88	10.46	21.88	10.50	22.00	10.51	22.18	10.65

Note: EDA, electrodermal activation; SCR, skin conductance response; TOTAL, mean of combined rejection types (devaluing, interruption, and nonverbal).
